# Determinants of female sexual orgasms

**DOI:** 10.3402/snp.v6.31624

**Published:** 2016-10-25

**Authors:** Osmo Kontula, Anneli Miettinen

**Affiliations:** Population Research Institute, Family Federation of Finland, Helsinki, Finland

**Keywords:** female orgasm, masturbation, determinants of orgasm, gender differences, sexual trends, communication, self-esteem, sexual desire, sexual techniques, good relationship

## Abstract

**Background:**

The pursuit of sexual pleasure is a key motivating factor in sexual activity. Many things can stand in the way of sexual orgasms and enjoyment, particularly among women. These are essential issues of sexual well-being and gender equality.

**Objective:**

This study presents long-term trends and determinants of female orgasms in Finland. The aim is to analyze the roles of factors such as the personal importance of orgasms, sexual desire, masturbation, clitoral and vaginal stimulation, sexual self-esteem, communication with partner, and partner’s sexual techniques.

**Design:**

In Finland, five national sex surveys that are based on random samples from the central population register have been conducted. They are representative of the total population within the age range of 18–54 years in 1971 (*N*=2,152), 18–74 years in 1992 (*N*=2,250), 18–81 years in 1999 (*N*=1,496), 18–74 years in 2007 (*N*=2,590), and 18–79 years in 2015 (*N*=2,150). Another dataset of 2,049 women in the age group of 18–70 years was collected in 2015 *via* a national Internet panel.

**Results:**

Contrary to expectations, women did not have orgasms that are more frequent by increasing their experience and practice of masturbation, or by experimenting with different partners in their lifetime. The keys to their more frequent orgasms lay in mental and relationship factors. These factors and capacities included orgasm importance, sexual desire, sexual self-esteem, and openness of sexual communication with partners. Women valued their partner’s orgasm more than their own. In addition, positive determinants were the ability to concentrate, mutual sexual initiations, and partner’s good sexual techniques. A relationship that felt good and worked well emotionally, and where sex was approached openly and appreciatively, promoted orgasms.

**Conclusion:**

The findings indicate that women differ greatly from one another in terms of their tendency and capacity to experience orgasms. The improvements in gender equality and sexual education since the 1970s have not helped women to become more orgasmic. Neither has the major increase in masturbation habits (among women in general). One challenge for future studies is to understand why women value their partner’s orgasms more than their own.

The pursuit of sexual pleasure is a key motivating factor in sexual activity. An orgasm is an effective indicator of sexual pleasure and healthy sexuality. In addition, orgasms are important predictors of happy relationships, and related sexual satisfaction. Without a doubt, a better understanding of the predictors of female sexual orgasms would be a most valuable achievement, and would be one key factor in improving equality among women, and gender equality (World Association for Sexual Health, 2014).

Previous studies have provided consistent results that men experience orgasms in intercourse considerably more frequently than women. More than 90% of men usually experience orgasm in their intercourse; among women, this proportion is only around 50% (Darling, Haavio-Mannila & Kontula, 2001; Kontula, 2009). This is a problematic observation from the perspective of both sexual rights and sexual health frameworks. Given the importance of orgasms to many people’s sexual health and pleasure, an increased focus on and understanding of women’s orgasm is valuable.

Although women have had more trouble than men in reaching orgasm, subjective descriptions of the event do not differ between genders (Meston, Levin, Sipski, Hull & Heiman, 2004).

The mental dimension of experiencing an orgasm seems very similar for both sexes. Meston, Levin, et al. (2004) reported that different studies have measured the duration of the female orgasm to be approximately 20–35 s. Both orgasm and vaginal stimulation have pain-suppressing effects (Komisaruk, Beyer-Flores & Whipple, 2006; Meston, Levin, Sipski, Hull & Heiman 2004).

Research has found that the capacity to experience orgasm during intercourse, and to a lesser extent in masturbation, is partly genetically determined (Dawood, Kirk, Bailey, Andrews & Martin, 2005; Dunn, Cherkas & Spector, 2005). An individual’s response to sexual pleasure during their life is a mixture of both the physical processes and the subjective responses to those processes. Some refer to the sensation of orgasm as being frightening; others speak of it as being the most exciting, fulfilling, and enjoyable sensation imaginable (Blackledge, 2004). Some women experience orgasm as the ultimate loss of control and consider it to be a vulnerability that should be avoided (Laan & Rellini, 2011).

It is sometimes suggested that orgasms may not be important for female sexual pleasure (Blackledge, 2004). The argument has been that women can be fully satisfied sexually without experiencing an orgasm. However, based on previous sex surveys, the most important single predictor of sexual satisfaction for women is without a doubt the orgasm (Kontula, 2009). If a woman did not have an orgasm in the latest intercourse, even 38% of women did not consider that intercourse pleasant. If they had an orgasm (or more than one), only a few women did not rate their intercourse as pleasant. This result concretely illustrates how crucial the role orgasms play in women’s assessment of the quality of sex they are having.

Female orgasms are also important for men. Men enjoy their partners’ orgasms, and they feel that they have the physical responsibility to stimulate their female partner to orgasm (Salisbury & Fisher, 2014). Opperman, Braun, Clarke, and Rogers (2014) found that both female and male participants felt responsible for their partner’s pleasure and ultimately their orgasm and, reciprocally, that their partners felt responsible for theirs. The most common concern reported by both male and female participants in Salisbury’s and Fisher’s study (2014) in regard to the lack of a female orgasm in sexual interaction focused on the male partner’s judgment of himself as a lover and the associated negative impact that the lack of a female orgasm would have on his self-esteem. Male participants reported judging themselves negatively if they were unsuccessful in their attempts to produce a female orgasm. Better knowledge of the predictors of female orgasms could therefore promote sexual well-being in both partners.

Finland is one of few countries with nationally representative surveys of sexual activities and values among the adult population. According to many international indicators, social progress is well advanced in Finland. In relation to social well-being, the European Quality of Life Survey gave the highest rates of happiness in Europe to Finland and Denmark. This has some implications for sexual values and activities. Sexual images and values are evolving to reflect a more affirming and liberal approach to sexuality.

Women have a unique position in Finland in international comparison. An important enduring element in Finnish society is the equal- and independent position of women. This can be seen in the realm of politics, education, paid work, and the division of labor in the home. The rate of women working full time in Finland is the highest in Western Europe – partly thanks to the extensive public childcare that is the right of every child.

Based on the Gender Equity Index (GEI), introduced by Social Watch, Finland is number one in the world in gender equity, along with Sweden. In education, Finland has been several times number one in the world regarding the results of the Programme for International Students Assessment (PISA) evaluation (60 countries) and OECD. Women outnumber men in higher education, and comprehensive sex education is at the highest level in Europe (Kontula, 2010).

Women’s right to be the initiators of sexual interactions was supported by 94% of Finnish men and 90% of women already in 1992 (Kontula & Haavio-Mannila, 1995). This support for female sexual autonomy has only increased since then (Kontula, 2009). This social and educational progress has created positive circumstances for sexual activities also among the aging population (Kontula, 2013).

During the last four decades, there have been major shifts in Finnish sexuality. Two nationally representative surveys of sexual behavior and sexual attitudes carried out in 1971 and 1992, showed that people’s attitudes have become more liberal; sexual behavior more equal; women sexually more active; and women’s sexual satisfaction in particular had increased during the 20 years between these two surveys. One of the main causes of this positive change (Kontula & Haavio-Mannila, 1995; Kontula & Kosonen, 1996) is estimated to have been the increasingly copious, open, and versatile treatment of sexuality in various media sources.

Over the last 20 years, the key shift in sexual culture in the West has been the opening up of the private sexual sphere into something that is now part of the public sphere (Kontula, 2009). This is manifested in the public proliferation of images of scantily clad people, intimate stories about well-known celebrities and personalities, and new technological breakthroughs in pornography. Sex and nudity are a natural and everyday part of public media culture. Sex is for everyone, even though not everyone has an equal opportunity to engage in it.

In many respects, sexual trends in Finland before the 2000s correspond to research data compiled previously in Europe on the same topic (Sandfort, Hubert, Bajos & Bos, 1998). The broader shift that has occurred in the West has meant a greater number of sexual partners before forming a committed relationship; lower levels of commitment in relationships; increased masturbation; an increase in lifetime partners and parallel relationships; and increasing commonness of oral and anal sex.

## Objective

The aim of this article is to present the predictors of one of the greatest present-day challenges in sexual life in Finland, namely female orgasms. This study includes long-term trends, and the determinants of female orgasms. The aim is to analyze various factors associated in female orgasms, including personal importance of orgasms, sexual desire, masturbation, clitoral and vaginal stimulation, sexual self-esteem, communication with partner, and partner’s sexual techniques.

## Methods

### Design

In Finland, five national FINSEX sex surveys, based on random samples from the Central Population Register, have been conducted, so that all Finns have had an equal opportunity to be selected into the sample. Respondents are representative of the total population within the age range of 18–54 years in 1971 (*N*=2,152), 18–74 years in 1992 (*N*=2,250), 18–81 years in 1999 (*N*=1,496), 18–74 years in 2007 (*N*=2,590), and 18–79 years in 2015 (*N*=2,150). In total, these surveys involve 10,637 respondents, 4,482 men and 6,155 women. The basic aim of these sex surveys has been to follow trends regarding a number of sexual issues.

The response rates were 91% (1971), 76% (1992), 46% (1999), 43% (2007), and 36% (2015). The higher response rates in 1971 and 1992 were due to the face-to-face interviews carried out at respondents’ homes. In 1999, 2007, and 2015, the data collection was carried out by Statistics Finland as a mailed survey (because of lower costs), which resulted in lower response rates.

The impact of the lower response rates in the 1999 and 2007 studies, as compared to the 1971 and 1992 surveys, has been evaluated by analyzing the ways in which people of particular birth cohorts have responded to the same questions concerning their own youth. The representativeness and comparability of the later data in relation to the 1992 data remained quite good, except in the case of male respondents over the age of 55. The later 1999 and 2007 findings provide a slight underestimation of male sexual activity over the age of 55 (sexual initiation somewhat later, and sexually a bit more monogamous in their life time), compared with the previous similar male cohorts of the respondents. Among women, a similar selection bias was not found. Data for 1999–2015 have been weighted to correct for the response bias.

More detailed information on the sampling, interviewing and questionnaires is available in Kontula & Haavio-Mannila (1995), Haavio-Mannila, Kontula & Kuusi (2001), Haavio-Mannila and Kontula (2003), and Kontula (2009).

Another data set (ORGSEX) of 2,049 women between 18–70 years of age was collected in May 2015 *via* a national Internet participant pool that includes 50,000 respondents. Sexual pleasure and orgasms were the core measures in this survey. The survey was conducted by Taloustutkimus Oy – Computer Aided Web Interview. Data were weighted as being representative of the whole population of Finland.

### Measures

The FINSEX questionnaires contained more than 100 questions, and many of them had a number of sub-sections. The following questions (translated from Finnish) selected from the questionnaire, specifically referred to orgasms in women:

How old were you when you reached orgasm through masturbation?

How old were you when you had an orgasm for the first time during sexual intercourse?

Do you usually have an orgasm during sexual intercourse?

Did you have an orgasm during your most recent sexual intercourse?

1. Including two or more orgasms.

By what type of activities do you usually experience orgasms during sexual intercourse?

1. By stimulating clitoris
2. By stimulating vagina
3. By stimulating them both

The ORGSEX questionnaire involved 21 questions and included some social background questions. Questions referring to measures of orgasm comprised:

Do you have orgasm during sexual intercourse?

Did you have an orgasm during your latest sexual intercourse?

1. Including two or more orgasms.

Do you have an orgasm more easily *via* masturbation or *via* intercourse?

Do you orgasm while you are stimulated *via* oral sex?

In what sexual position do you most easily have an orgasm?

What helps you the most to have an orgasm?

1. Strong personal arousal
2. Partner’s strong arousal
3. Favorable undisturbed and erotic situation
4. Using enough time for love making
5. A skillful and attractive partner
6. Oral or manual stimulation
7. Use of a sexual toy or massage-machine
8. Can’t tell

What issues prevent you from having an orgasm during love-making?

1. Eight alternatives to select

How important do you consider it to be to have an orgasm in love-making?

How important do you consider it to be in love-making to provide an orgasm to your partner?

In what ways have you learned to intensely enjoy love-making and to experience orgasms?

1. Eight alternatives to select

The analyses were conducted using IBM SPSS Statistics Version 23. In addition to descriptive analysis (Figs. 1–14), we examined the main determinants of the female sexual orgasms with regression analyses (Tables 1–4). There were also a few chi-square tests.

**Fig. 1:**
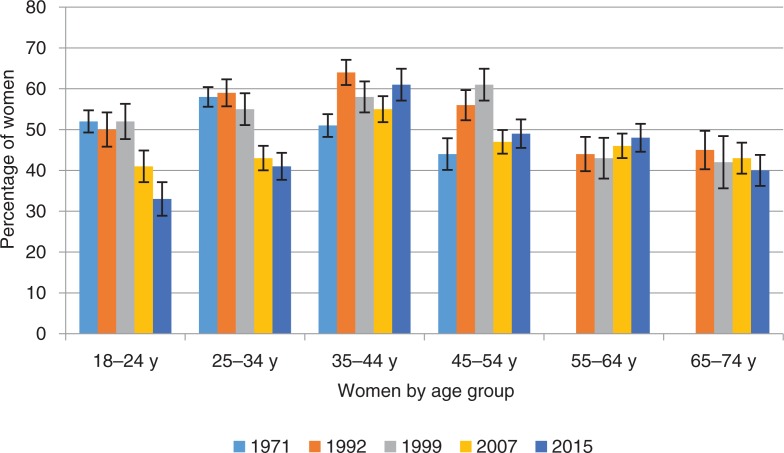
Percentage of women who experienced orgasm most of the time or always during sexual intercourse by age group and survey year. Intercourse defined as penile–vaginal intercourse. Error bars indicate standard errors. FINSEX 1971–2015.

**Fig. 2:**
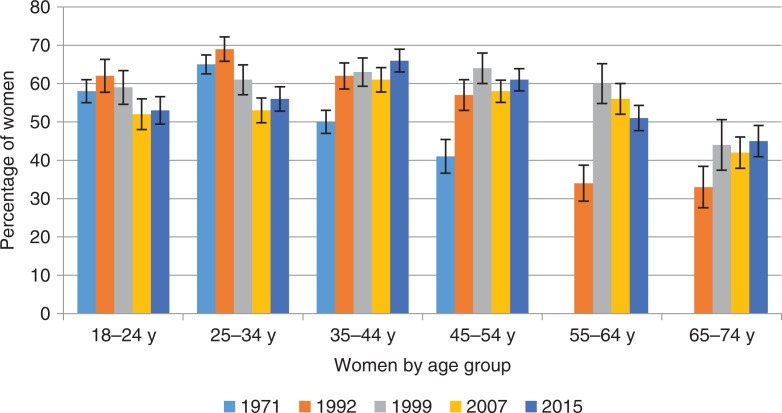
Percentage of women reporting orgasm during their last intercourse by age group and survey year. Intercourse defined as penile–vaginal intercourse. Error bars indicate standard errors. FINSEX 1971–2015.

**Fig. 3:**
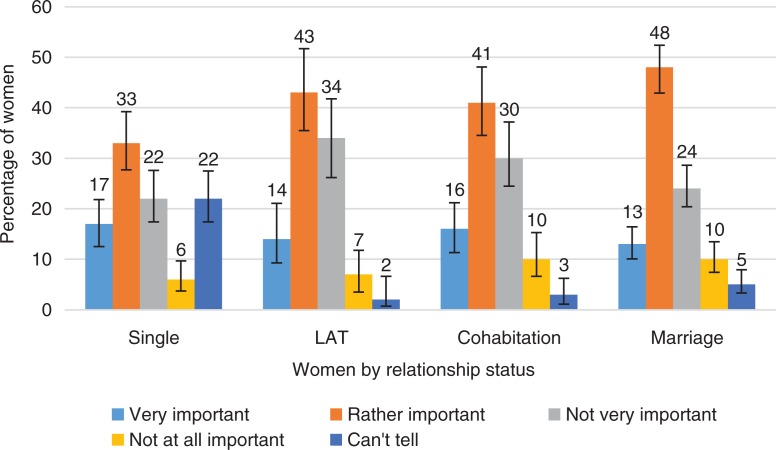
Importance of experiencing orgasm during love-making for women in different relationship statuses. LAT=Living-apart-but-together (i.e. in relationship but not cohabiting). Marriage includes persons living in registered unions. Definition of love-making left to the participant. Error bars indicate 95 CI. ORGSEX 2015.

**Fig. 4:**
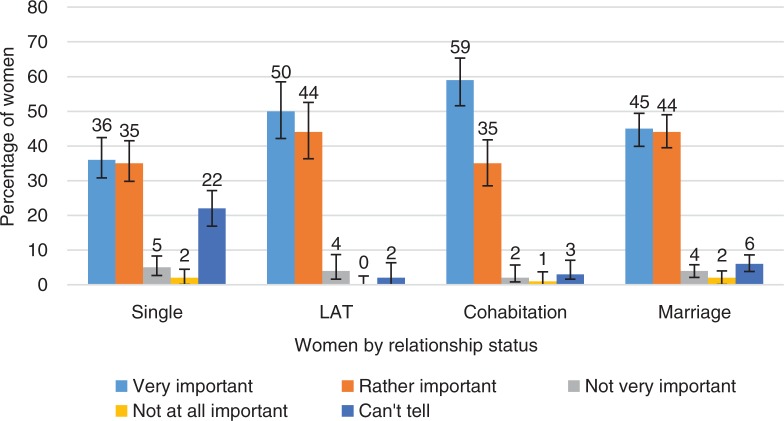
Women’s responses to question of how important it is to produce an orgasm in their partner during love-making. Women grouped by their relationship status. Exact wording of the question: ‘How important it is for you to produce an orgasm in your partner during love-making?’ Definition of love-making left to the participant. LAT=Living-apart-but-together (i.e. in relationship but not cohabiting). Marriage includes persons living in registered unions. Error bars indicate 95 CI. ORGSEX 2015.

**Fig. 5:**
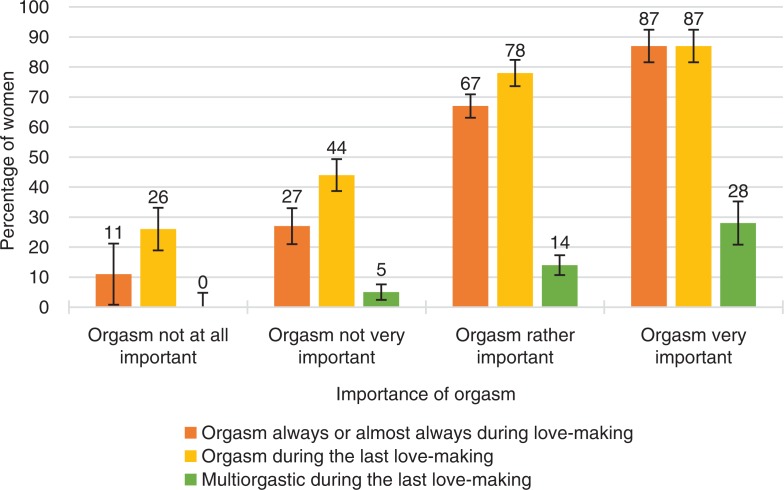
Women’s orgasmic capacity by how important orgasm is to them. Note that orgasmic capacity is here measured with three separate variables: (1) ‘Sexual pleasure ending in relaxation and a very good feeling is called an orgasm. Do you have an orgasm during love-making?’ The proportion of women who answered ‘Always’ or ‘Almost always or usually’ is depicted in the figure with the orange bar. (2) ‘Sexual pleasure ending in relaxation and a very good feeling is called an orgasm. Did you have an orgasm during your last love-making?’ The proportion of women who answered ‘Yes, one’ or ‘Yes, two’ or ‘Yes, more than two’ is depicted in the Figure with the yellow bar. (3) ‘Sexual pleasure ending in relaxation and a very good feeling is called an orgasm. Did you have an orgasm during your last love-making?’ The proportion of women who answered ‘Yes, two’ or ‘Yes, more than two’ is depicted in the Figure with the green bar. Definition of love-making is left to the participant. Error bars indicate 95 CI. ORGSEX 2015.

**Fig. 6:**
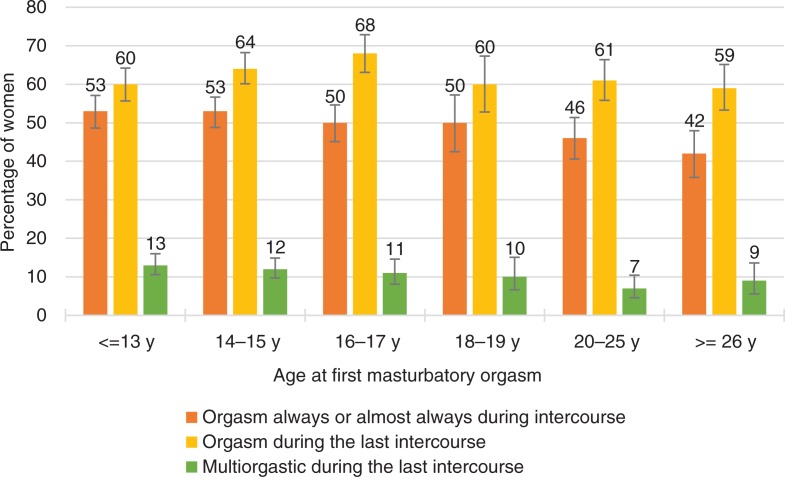
Women’s orgasmic capacity by the age at first masturbatory orgasm. Note that orgasmic capacity is here measured with three separate variables: (1) ‘Sexual pleasure ending in relaxation and a very good feeling is called an orgasm. Do you have an orgasm during intercourse?’ The proportion of women who answered ‘Always’ or ‘Almost always or usually’ is depicted in the figure with the orange line. (2) ‘Sexual pleasure ending in relaxation and a very good feeling is called an orgasm. Did you have an orgasm during your last intercourse?’ The proportion of women who answered ‘Yes, one’ or ‘Yes, two or more’ is depicted in the Figure with the yellow line. (3) ‘Sexual pleasure ending in relaxation and a very good feeling is called an orgasm. Did you have an orgasm during your last intercourse?’ The proportion of women who answered ‘Yes, two or more’ is depicted in the Figure with the green line. Intercourse defined as penile-vaginal intercourse. Error bars indicate 95 CI. FINSEX 1999–2015.

**Fig. 7:**
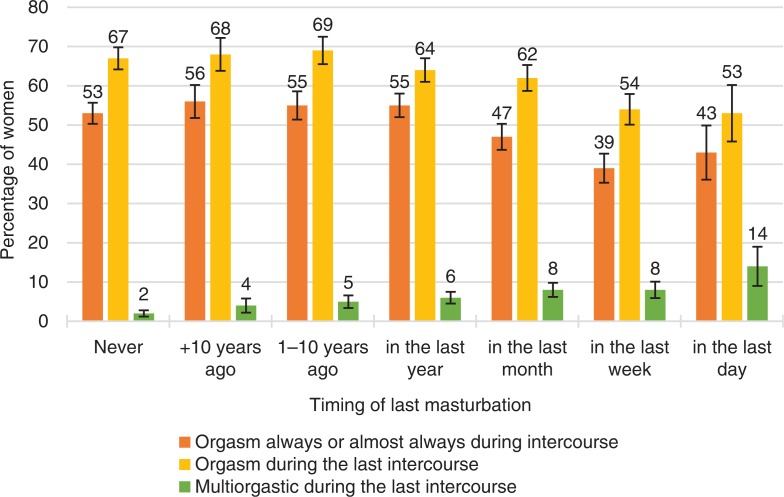
Women’s orgasmic capacity by timing of last masturbation. Note that orgasmic capacity is here measured with three separate variables: (1) ‘Sexual pleasure ending in relaxation and a very good feeling is called an orgasm. Do you have an orgasm during intercourse?’ The proportion of women who answered ‘Always’ or ‘Almost always or usually’ is depicted in the figure with the orange bars. (2) ‘Sexual pleasure ending in relaxation and a very good feeling is called an orgasm. Did you have an orgasm during your last intercourse?’ The proportion of women who answered ‘Yes, one’ or ‘Yes, two or more’ is depicted in the Figure with the yellow bars. (3) ‘Sexual pleasure ending in relaxation and a very good feeling is called an orgasm. Did you have an orgasm during your last intercourse?’ The proportion of women who answered ‘Yes, two or more’ is depicted in the Figure with the green bars. Intercourse defined as penile–vaginal intercourse. Error bars indicate 95 CI. FINSEX 1971–2015.

**Fig. 8:**
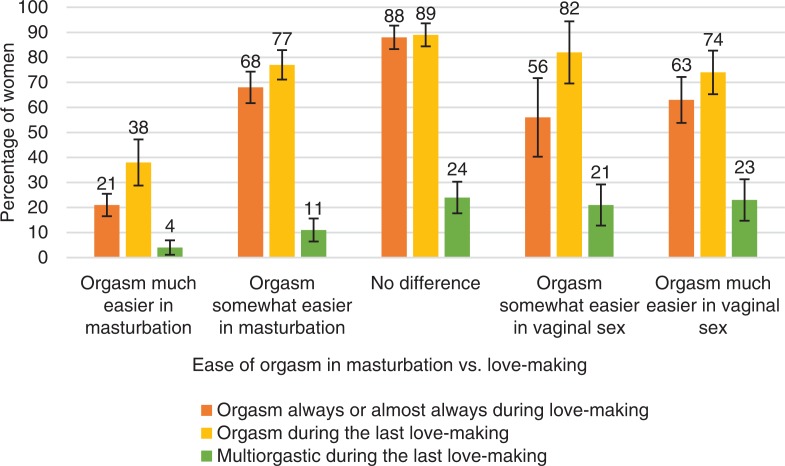
Women’s orgasmic capacity by whether orgasm is easier to achieve in masturbation or in love-making. Note that orgasmic capacity is here measured with three separate variables: (1) ‘Sexual pleasure ending in relaxation and a very good feeling is called an orgasm. Do you have an orgasm during love-making?’ The proportion of women who answered ‘Always’ or ‘Almost always or usually’ is depicted in the figure with the orange bars. (2) ‘Sexual pleasure ending in relaxation and a very good feeling is called an orgasm. Did you have an orgasm during your last love-making?’ The proportion of women who answered ‘Yes, one’ or ‘Yes, two’ or ‘Yes, more than two’ is depicted in the Figure with the yellow bars. (3) ‘Sexual pleasure ending in relaxation and a very good feeling is called an orgasm. Did you have an orgasm during your last love-making?’ The proportion of women who answered ‘Yes, two’ or ‘Yes, more than two’ is depicted in the Figure with the green bars. Definition of love-making is left to the participant. Error bars indicate 95 CI. ORGSEX 2015.

**Fig. 9:**
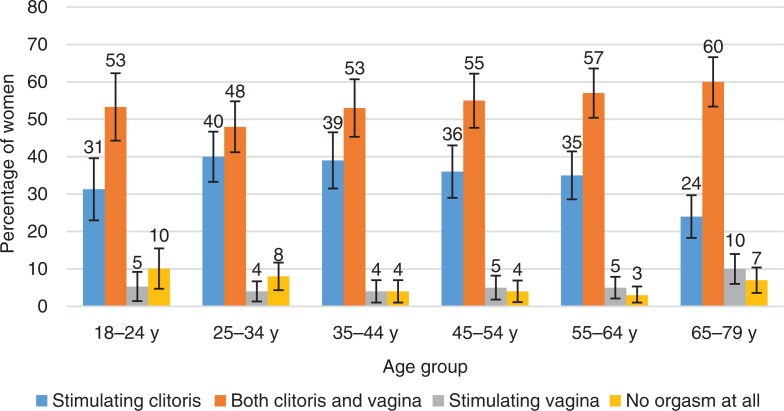
How women typically achieve orgasm during sexual interaction by age group. Exact phrasing of the question: ‘How do you usually achieve orgasm during sexual interaction?’ Sexual interaction defined as either intercourse, oral sex, or manual sex. Error bars indicate 95 CI. FINSEX 2015.

**Fig. 10:**
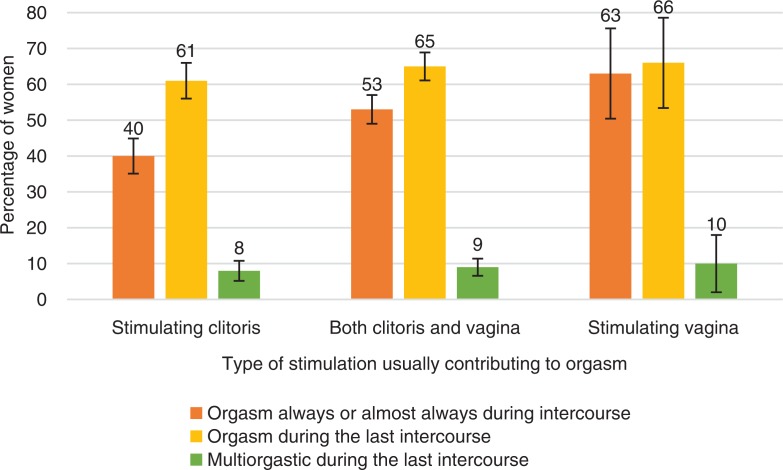
Women’s orgasmic capacity by the type of stimulation they report as usually contributing to orgasm. Note that orgasmic capacity is here measured with three separate variables: (1) ‘Sexual pleasure ending in relaxation and a very good feeling is called an orgasm. Do you have an orgasm during intercourse?’ The proportion of women who answered ‘Always’ or ‘Almost always or usually’ is depicted in the figure with the orange bars. (2) ‘Sexual pleasure ending in relaxation and a very good feeling is called an orgasm. Did you have an orgasm during your last intercourse?’ The proportion of women who answered ‘Yes, one’ or ‘Yes, two or more’ is depicted in the Figure with the yellow bars. (3) ‘Sexual pleasure ending in relaxation and a very good feeling is called an orgasm. Did you have an orgasm during your last intercourse?’ The proportion of women who answered ‘Yes, two or more’ is depicted in the Figure with the green bars. Exact phrasing of the question: ‘How do you usually achieve orgasm during sexual interaction?’ Sexual interaction defined as sexual intercourse, oral sex, or manual sex. Error bars indicate 95 CI. FINSEX 2015.

**Fig. 11:**
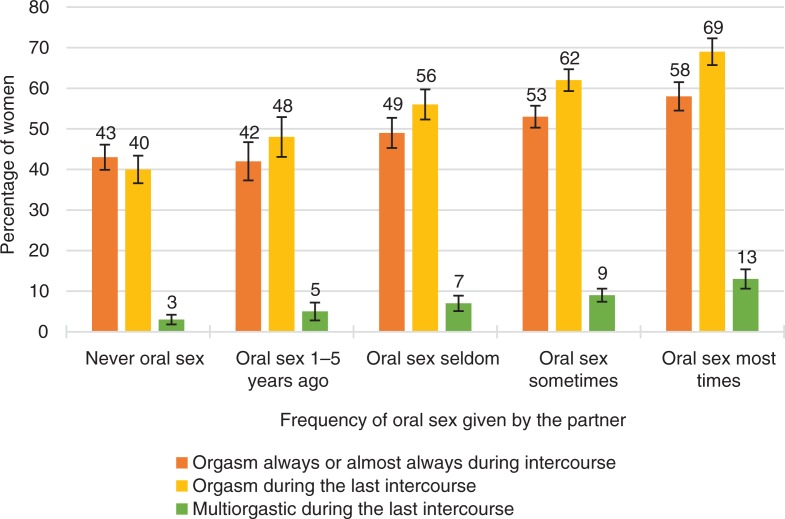
Women’s orgasmic capacity by how often partner has given oral sex. Note that orgasmic capacity is here measured with three separate variables: (1) ‘Sexual pleasure ending in relaxation and a very good feeling is called an orgasm. Do you have an orgasm during intercourse?’ The proportion of women who answered ‘Always’ or ‘Almost always or usually’ is depicted in the figure with the orange bars. (2) ‘Sexual pleasure ending in relaxation and a very good feeling is called an orgasm. Did you have an orgasm during your last intercourse?’ The proportion of women who answered ‘Yes, one’ or ‘Yes, two or more’ is depicted in the Figure with the yellow bars. (3) ‘Sexual pleasure ending in relaxation and a very good feeling is called an orgasm. Did you have an orgasm during your last intercourse?’ The proportion of women who answered ‘Yes, two or more’ is depicted in the Figure with the green bars. Exact phrasing of the question: ‘During intercourse, how often you have been given oral sex by your partner during past 5 years?’ Intercourse defined as penile–vaginal intercourse. Error bars indicate 95 CI. FINSEX 1992–2015.

**Fig. 12:**
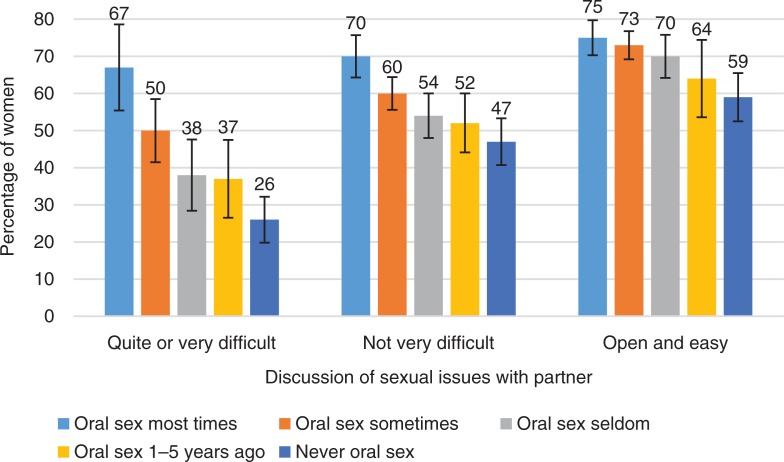
Percentage of women experiencing orgasm during the last intercourse by how often partner has given oral sex and by how easy or difficult it is to have discussions of sexual issues with one’s partner. Exact phrasing of the questions: ‘Is discussion of sexual issues easy or difficult with your partner?’; ‘During intercourse, how often you have been given oral sex by your partner during past 5 years?’ Intercourse defined as penile–vaginal intercourse. Error bars indicate 95 CI. FINSEX 1992–2015.

**Fig. 13:**
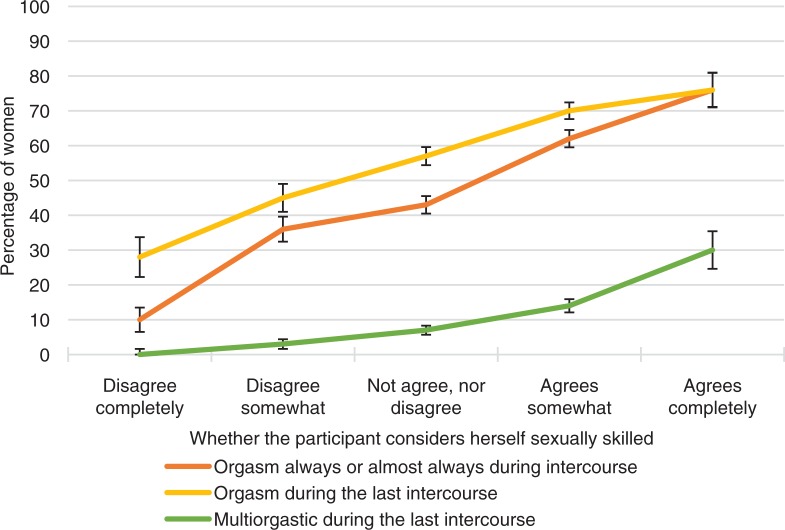
Women’s orgasmic capacity by whether they agree they are ‘sexually quite skilled’. Note that orgasmic capacity is here measured with three separate variables: (1) ‘Sexual pleasure ending in relaxation and a very good feeling is called an orgasm. Do you have an orgasm during intercourse?’ The proportion of women who answered ‘Always’ or ‘Almost always or usually’ is depicted in the figure with the orange line. (2) ‘Sexual pleasure ending in relaxation and a very good feeling is called an orgasm. Did you have an orgasm during your last intercourse?’ The proportion of women who answered ‘Yes, one’ or ‘Yes, two or more’ is depicted in the Figure with the yellow line. (3) ‘Sexual pleasure ending in relaxation and a very good feeling is called an orgasm. Did you have an orgasm during your last intercourse?’ The proportion of women who answered ‘Yes, two or more’ is depicted in the Figure with the green line. Exact phrasing of the question: ‘Do you agree or disagree with the following: I consider myself quite skilled in sexual issues’. Intercourse defined as penile–vaginal intercourse. Error bars indicate 95 CI. FINSEX 1992–2015.

**Fig. 14:**
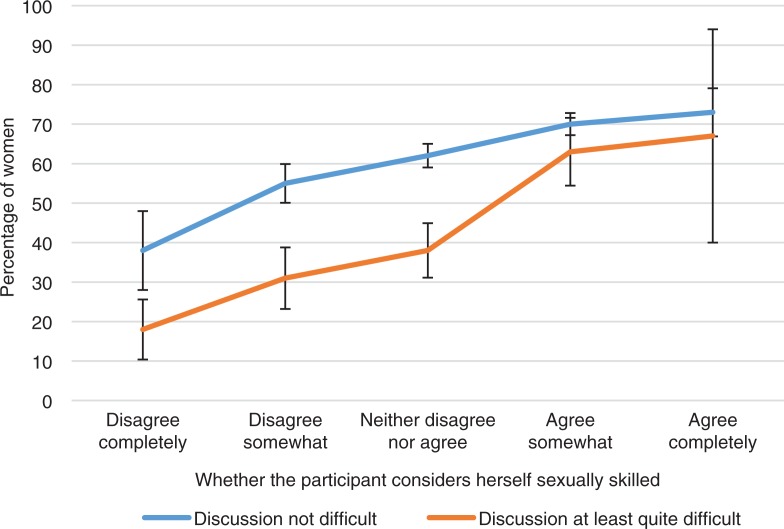
Percent of women achieving orgasm in the last intercourse by whether they agree they are sexually skilled and by ease of discussion of sexual issues with partner. Exact phrasing of the question: ‘Do you agree or disagree with the following: I consider myself quite skilled in sexual issues’. Question on easiness of discussion of sexual issues with partner collapsed into two categories: (1) at least quite difficult=quite or very difficult; (2) not difficult=not very difficult or open and easy. Intercourse defined as penile–vaginal intercourse. Error bars indicate 95 CI. FINSEX 1992–2015.

**Table 1:** Associations between socio-demographic, lifestyle and relationship history characteristics and orgasmic frequency in women

	Coeff	*p*	*N*
Socio-demographic			
Education (years in education)	−0.007	0.185	5477
Income	0.028***	0.000	5389
Importance of religion	0.083***	0.000	5420
Attendance of religious services	0.041*	0.015	3258
Sexual attitudes: Woman has a right to initiate sex	0.111***	0.000	5505
Physical exercise	0.039**	0.009	4120
BMI	0.008	0.131	5474
BMI squared	−0.001+	0.060	5474
Alcohol consumption (6=never)	0.001	0.872	5457
Heavy drinking (6=never)	0.034*	0.014	4192
Smoking (3=smokes)	0.047*	0.033	5500
Psychological symptoms (24=many symptoms)	−0.038***	0.000	5179
Psychological distress, anxiety (3=often)	−0.203***	0.000	5339
Couple relationship characteristics & history			
Number of steady relationships (1+)	0.057+	0.091	4578
Number of persons fallen in love with (0+)	0.015	0.301	5403
Number of sex partners within last 5 years (0+)	−0.001	0.813	3071
Number of sex partners lifetime (1+)	0.000	0.816	5289
Has had extra-marital relationship(s) (1=no)	0.045	0.465	4249
Current relationship: married (vs. single)	0.476***	0.000	5529
Current relationship: cohabiting (vs. single)	0.422***	0.000	5529
Current relationship: LAT (relationship, not cohabiting) (vs. single)	0.399***	0.000	5529
Duration of current relationship	−0.002	0.479	4422
Children: own children (1=has children)	0.408***	0.000	5509
Children: household children (1=lives children)	0.214***	0.000	4243
Importance of orgasm and sexual satisfaction			
Importance of sex for the couple relationship (4=very important)	0.445***	0.000	4382
How important to have orgasm in love-making (4=very important)	1.026***	0.000	952
How important to provide orgasm to partner (4=very important)	0.573***	0.000	953

+=*p*<0.1;

* *p*<0.05;

** *p*<0.01;

*** *p*<0.001.

Coefficients are from regression models adjusting for age and survey year.

**Table 2:** Associations between sexual experiences, sexual skills and couple relationship characteristics with orgasmic frequency in women

	Coeff	*p*	*N*
Intercourse and masturbation, lifetime			
Age at first intercourse	−0.028***	0.000	5460
Age at first orgasm in intercourse	−0.044***	0.000	4925
Frequency of intercourse during past month (8=daily)	0.165***	0.000	5476
Recent masturbation (7=during last 24 h)	−0.091***	0.000	5310
Frequency of masturbation (6=4+ times/w)	−0.036	0.414	955
Age at first orgasm in masturbation	−0.005	0.203	2313
Experiences related to intercourse			
Partner comes too quickly (4=frequently)	−0.308***	0.000	3658
Intercourse feels painful (4=frequently)	−0.365***	0.000	1723
Experiences: vaginal dryness (6=constantly)	−0.141***	0.000	3697
Experiences: partner has erection problems (6=constantly)	−0.111***	0.000	4653
Sex techniques and skills			
Orgasm *via* clitoris or vagina or both: vagina (vs. clitoris)	0.640***	0.000	1065
Orgasm *via* clitoris or vagina or both: both (vs. clitoris)	0.518***	0.000	1065
Duration of intercourse	0.207***	0.000	2456
Partner gives oral stimulation (5=most times)	0.154***	0.000	4152
Last time since partner gave manual stimulation (7=less than a week)	0.170***	0.000	1071
Lack of own sexual desire (4=never)	0.353***	0.000	3826
Sexual self-esteem (5=agree completely)	0.573***	0.000	953
Orgasm much easier *via* masturbation (vs. much easier *via* love-making)	−1.585***	0.000	848
Somewhat easier *via* masturbation (vs. much easier *via* love-making)	−0.077	0.559	848
As easy in masturbation as in love-making (vs. much easier *via* love-making)	0.473***	0.002	848
Somewhat easier in love-making (vs. much easier *via* love-making)	−0.259	0.254	848
Couple relationship: satisfaction and communication			
Satisfaction towards couple relationship (4=very happy)	0.332***	0.000	4412
Discussion of sexual issues with partner (4=open and easy)	0.370***	0.000	4405
Physical intimacy in the couple relationship (4=not at all)	−0.454***	0.000	3476

+=*p*<0.1;

* *p*<0.05;

** *p*<0.01;

*** *p*<0.001.

Coefficients are from regression models adjusting for age and survey year.

**Table 3:** Determinants of female orgasmic capacity: FINSEX-data

	2007–2015		1992–2015		1992 and 2015	
			
Survey years	Coeff	*p*	Coeff	*p*	Coeff	*p*
Frequency of intercourse (during past 1 month)	−0.034	0.142	−0.023	0.165	−0.038	0.127
Duration of intercourse	0.124***	0.000	–	–	–	–
Intercourse feels painful	–	–	–	–	−0.174	0.000
Age at first intercourse	−0.028*	0.013	−0.030***	0.000	−0.030**	0.002
Duration since last masturbation	0.087***	0.000	0.087***	0.000	0.102***	0.000
Partner gives oral stimulation	0.014	0.605	0.034	0.079	0.009	0.742
Partner comes too quickly	−0.236***	0.000	−0.289***	0.000	−0.339***	0.000
Sexual self-esteem	0.223***	0.000	0.200***	0.000	0.185***	0.000
Sex important for happiness in couple relationship	0.226***	0.000	0.285***	0.000	0.288***	0.000
Satisfaction towards couple relationship	0.126***	0.005	0.130***	0.000	0.055	0.257
Discussion of sexual issues with partner easy	0.119***	0.009	0.130***	0.000	0.153**	0.003
*N*	1682		2910		1353	

+=*p*<0.1;

* *p*<0.05;

** *p*<0.01;

*** *p*<0.001.

Model include controls for education, religiousity, age, and survey year.

**Table 4:** Determinants of female orgasmic capacity: ORGSEX-data

	Coeff	*p*
Frequency of intercourse (during past 1 month)	−0.001	0.970
Duration of intercourse	0.098***	0.001
Sexual self-esteem	0.289***	0.000
Frequency of masturbation	0.040	0.360
Achieving orgasm in love-making important	0.854***	0.000
Orgasm easier *via* love-making than *via* masturbation	0.296***	0.000
*N*	985	

+=*p*<0.1;

* *p*<0.05;

** *p*<0.01;

*** *p*<0.001.

Model include controls for age.

## Results

### Trends in female orgasms

A major challenge in Finnish sexuality is the declining trends in female sexual satisfaction and orgasm. For women, having an orgasm from intercourse is much less guaranteed than for men. In 2015, 46% of women said that they always or nearly always had an orgasm when having intercourse, with only 6% of women reporting always having an orgasm. Nearly one in six (16%) women had an orgasm approximately half of the time and 38% of women had orgasms fairly infrequently at most. Over 16 years (1999–2015), women’s orgasmic capacity has declined considerably, from 56% of women experiencing orgasm in intercourse always or nearly always in 1999 to 46% in 2015 (χ^2^(20)=84.8, *p*=0.000). The decline is apparent among both young and middle aged women. In similar fashion, the proportion of women who have had an orgasm in the latest intercourse has diminished from 1999.

Difficulties experiencing orgasms has affected a large proportion of women. In 2015, 9% of women reported never having had an orgasm from intercourse. In earlier studies, the proportion of women who had never experienced an orgasm from sexual intercourse was 4–7%, which is lower than in 2015. Furthermore, according to the 2015 findings, 14% of young women (under the age of 35) in particular had never had an orgasm from intercourse. This is a higher figure than in previous surveys.

It is of particular note that in 2015, only 38% of young women (vs. 42% in 2007 and 53% in 1999) reported that they usually had an orgasm during intercourse, whereas 43% said that they had an orgasm fairly infrequently at most. Similar proportions were observed in the ORGSEX survey, in which ‘love-making’ was the adopted concept instead of intercourse. Again, only 38% of women aged 18–24 ‘usually’ had an orgasm in love-making. In previous surveys, middle-aged and older women up to retirement-age reported a higher incidence of orgasms than women in younger age groups, and there is a similar trend nowadays. In the framework of sexual well-being and sexual health, decline in orgasmic capacity is a major sexological challenge in the 2000s.

The proportion of people who had an orgasm the last time they had intercourse was close to the proportion of women who said that they generally had an orgasm from intercourse. Of the women surveyed in 2015, 54% had had an orgasm the last time they had intercourse. However, there was also some confusion surrounding what an orgasm is or should be. A total of 6% of women were not able to tell whether or not they had had an orgasm the last time they had intercourse.

These findings indicate that women differ greatly from one another in terms of their tendency or capacity to experience orgasms. A significant proportion of women (19%) experienced persistent problems experiencing any orgasm from intercourse, whereas many (8% of all women and 11% of young women) found it easy to have multiple orgasms. The variation in sexual enjoyment among women was drastically greater than among men. It is particularly intriguing that women are now experiencing greater, not fewer, problems regarding orgasms as compared to past decades, even though the opportunities for gender equality and sexual enjoyment in society now seem to be better than ever before.

### First experiences of orgasms

Most young women experience their first orgasm during masturbation. In 2015, half of the youngest generation of women (under the age of 35) had experienced their first orgasm in masturbation before the age of 15. The age of first orgasm achieved *via* masturbation was in steady decrease from the oldest generation (over the age of 55) to the youngest generation. The average age at first orgasm in masturbation declined significantly from 22 years (age group 65+ years) to 15 years (age group 18–24 years). The average age had fallen in the 2000s by 3.5 years among young women in comparison with the oldest age group. In the oldest generation, only one-tenth of women had experienced their first orgasm in masturbation before the age of 15. There was a continuous declining trend in this age from one survey to the next and from one generation to another (*r*=0.365, *p*=0.01).

In 2015, 2015a quarter of young women had their first orgasm in masturbation before the age of 13 and one-tenth before the age of 10. Some women reported that they had their first orgasm in masturbation as early as the age of 5. However, many women had not experienced an orgasm until they were in their 40s or 50s. The oldest reported ages of participants experiencing their first orgasm *via* masturbation were women in their 60s. At the population level, there seems to be a huge variation in the age of first orgasm in masturbation.

Women are significantly increasing their rate of masturbation over time, and across surveys (Kontula, 2009). Although masturbation provides women with a lot pleasure, orgasms from intercourse have been found to be more pleasurable. In Mah and Binik’s (2002) study both men and women recall orgasms experienced with a partner present as having been significantly more pleasurable and satisfying than those occurring during solitary masturbation.

The trends in women’s first orgasms achieved during intercourse are very different from their first orgasms achieved *via* masturbation. To some, it may be surprising how large the discrepancy is between some women’s age at first intercourse, and their first time experiencing orgasm through intercourse. Although women had their first intercourse, on average, at the age of 17, only a third of women had their first orgasm at intercourse under the age of 18. A quarter of women, but three quarters of men, had achieved an orgasm in the same year as their first intercourse. Altogether 40–50% of women had their first orgasm at intercourse only after the age of 20.

This proportion has remained quite stable since the 1992 survey. In addition, the average age of first orgasm during intercourse was similar in older and younger generations, namely around 20–21 years of age. The outcome was that the difference between the age at first orgasm in intercourse, and the age of the first orgasm in masturbation had increased. For women, it was common that there was a few years’ gap between the time of their first intercourse, and the time of their first experience of orgasm in intercourse.

Most women have had their first orgasm during masturbation. Half of the women surveyed had their first orgasm during masturbation at least 5 years before their first orgasm during intercourse, and 17% of women 10 years before their first orgasm in intercourse. Only about 10% of women had their first orgasm during intercourse before experiencing an orgasm *via* masturbation.

The implication of these results is that women have usually been able to practice their orgasms for several years before experiencing them for the first time in intercourse. It has been hypothesized that this type of physical practice should enable them to achieve orgasms in intercourse more easily (McCabe, 2009). However, in these data, this expected positive outcome did not exist (Table 2).

### Determinants of female orgasms

#### Poor determinants of female orgasms

We examined the association between several socio-demographic, lifestyle and personal characteristics and orgasmic capacity in the pooled FINSEX data. Due to the large data set, the associations often proved to be statistically significant. However, in many cases the differences in the ability to experience orgasms between different groups of women were relatively small. The coefficients from the regression analyses and *p*-values are reported in Table 1, and in the following, we focus on those results which appeared to influence women’s orgasmic capacity the most. Women’s social background was only weakly associated with the ability to experience orgasms. Women had orgasms almost with the same frequency, regardless of their education or income levels. On the other hand, religious women were more likely to experience orgasms in the intercourse than were those women who regarded religion not at all important. The association was much weaker when church attendance was considered. Somewhat contrasting, more self-determining attitudes toward sexuality issues (‘woman has the right to make sexual initiatives’) were also associated with higher orgasmic capacity.

There were a number of other lifestyle and personal characteristics that were not associated or only very weakly associated with the frequency of orgasm. These factors included physical exercise, psychological symptoms, smoking, and moderate alcohol use. On average, 46% of women with BMI below 20 experienced orgasm always or almost always during intercourse, compared to 51% among normal or slightly overweight women, or 50% among obese women (age-adjusted figures). Mild mental health problems were not linked to the problem of having orgasms, while constant feelings of anxiety and distress were associated with decreased likelihood of experiencing orgasms.

#### Relationship and sexual partner history

Women’s relationship and sexual partner history appeared to have no effect on the ability of women to have orgasms (Table 1). Women’s orgasm frequency did not vary according to the number of steady relationships that they had had in their lifetime, nor did it vary according to the number of times in life they had fallen in love. The same was also true regarding the number of sexual partners in recent years, or over their lifetime. In addition, women’s ability to achieve an orgasm was not associated with being unfaithful at some point in their current relationship.

However, sexual experience with a steady partner was positively associated with the frequency of having orgasms. Only 40% of single women usually experienced orgasms in intercourse when the respective figure for women in marital, cohabiting or living apart together (LAT)-unions was above 50%. Women who were in newer relationships of only a few years at most had more frequent difficulties achieving orgasms than other women. This was partly related to their young age, and the effect of union duration disappeared once we controlled for the age.

#### How important orgasms were considered

The ORGSEX survey asked questions about how important women considered attaining an orgasm themselves to be in love-making, and how important they felt it was to produce an orgasm to their own partner. The results are presented by women’s relationship status (Figs. 3 and 4).

Around 60% of women considered having an orgasm at least ‘rather important’ in love-making, though less than 20% rated it as ‘very important’. Additionally, 10% of women thought that an orgasm was ‘not at all important’ in love-making. In fact, they usually rated their partner’s orgasm to be more important than their own.

Almost all women said that it was at least ‘rather important’ to bring their partner to orgasm. Half of women considered their partner’s orgasm ‘very important’. This proportion was much higher than the proportion of women considering their own orgasm to be ‘very important’. In Nicholson and Burr’s (2003) study, women reported that it was important to ‘give’ their male partners pleasure and orgasm, possibly at the expense of their own pleasure. In Salisbury’s and Fisher’s study (2014) women asserted that their orgasm was more of a ‘bonus’ than a goal of sexual interactions.

Only single women valued orgasms differently (they more often could not tell how important their partner’s orgasm might be), but even they consider a future partner’s orgasm more important than their own. Based on these results, women were in their sexual interaction quite altruistic – at least according to them. Two-thirds of the women who thought that their orgasm was not at all important considered their partner’s orgasm at least rather important.

Evaluation of women’s own orgasms in love-making was highly associated with their orgasmic capacity. Of the women who considered their orgasms to be very important, almost 90% usually had orgasms during intercourse, and also had one in their latest intercourse (Fig. 5). These results are in line with Laan and Rellini (2011) who determined that women who found it easier to orgasm were also more likely to regard orgasm as important. Of the women who considered their orgasms very important, almost 30% had also a multi-orgasmic experience in their latest intercourse. This association may be partly explained by highly pleasurable sexual experiences prompting women to place a higher value on orgasms.

At the other end of the orgasm-evaluation scale, were women who did not consider their orgasms to be important. Most of them had difficulties experiencing orgasms. Only 13% of these women had an orgasm in their latest intercourse. Because these women rarely experienced orgasm, it makes sense that they did not value orgasms that much in their love-making. Laan and Rellini (2011) note that a low female value on own orgasm can be considered a sensible coping strategy, in that, by placing less value on orgasms if they are difficult or impossible to have, they will not be disappointed by their sexual experiences.

Female orgasmic capacity was also related to how important women considered sex to be for the happiness of their current relationship. If they considered sex to be important or very important for the happiness, they reported having had an orgasm in their latest intercourse more often, and were more likely to usually achieve orgasm in intercourse (Table 1, *p*<0.001). They reported having experienced an orgasm even more often if they also rated their relationship as being happy. Of women who were very happy in their current romantic relationship, and who also considered sex very important for happiness in a relationship, 76% reported having had an orgasm in their latest intercourse. If they did not value sex highly in their relationship, and they had a relationship that was not happy, only 29% reported orgasmic experiences in the most recent intercourse. Happy relationships were associated with orgasm capacity (Table 1, *p*<0.001), but less so if women did not consider sex to be important to the happiness of their current relationship.

#### Orgasms and sexual techniques

In sexual therapy, a common assumption has been that physically practicing masturbation or sexual pleasuring will increase a women’s ability to experience orgasms in intercourse.

These two sexuality survey data (FINSEX and ORGSEX) did not provide clear support for this assumption. The age at which women began to have sexual intercourse was statistically significantly associated with the overall ability to experience orgasms during sexual intercourse (Table 2, *p*<0.001). Among early initiators (first intercourse by age 17) 53% of women had orgasms almost always during the intercourse, among women who had had their first intercourse at later age (18–24 years), the respective figure was 47%. Of women who had their sexual initiation after age 25, 44% were able to usually have orgasm during the intercourse.

On the other hand, age at which women first had an orgasm in masturbation was not statistically significantly related to orgasmic capacity. This was true also regarding if the women had one orgasm or several orgasms in their latest intercourse. In addition, masturbation frequency was negatively (not statistically significantly) associated with how often women experienced orgasm during intercourse. In fact, those women who had masturbated never or only a very long time ago, were more likely to experience orgasms during intercourse (Table 2, *p*<0.001).

One masturbation-related assumption is that women achieve orgasm *via* masturbation more easily than *via* intercourse. These data provide some confirmation of this hypothesis. Nearly half of women (48%) reported that they achieved orgasm more easily in masturbation than in intercourse. However, 14% of women achieved an orgasm more easily *via* intercourse than masturbation, while 17% achieved it similarly easily in both ways, and 20% of all women could not tell by which technique they found it easier to have orgasms.

The ease of achieving an orgasm *via* masturbation versus intercourse had no clear association with how often women had experienced orgasms in intercourse, or if they had had an orgasm in their latest intercourse. There was a low frequency of orgasm in intercourse among women who much easier achieved orgasm *via* masturbation (only 38% of them had an orgasm in the latest intercourse), this association was statistically significant (*r*=0.37, *p*<0.001). On the other hand, there was hardly any differences in the orgasmic capacity between women who achieved orgasm more easily *via* vaginal sex as compared to women who achieved orgasm somewhat more easily *via* masturbation. Women who could orgasm equally easily both *via* masturbation and vaginal sex were the most orgasmic in the latest intercourse (90%) (Fig. 8).

A continuous international debate has been if women achieve orgasm more easily *via* stimulating their clitoris or *via* stimulating their vagina (Paget, 2001). Paget continues that the discussion can follow the spirit of Masters and Johnson regarding clitocentrism, including that a woman can orgasm only *via* clitoral stimulation.

Blackledge (2004) tells that sexual arousal typically occurs as a result of the activation of various nerves. Typically when orgasm occurs, it is the result of one or more of three genital nerves being activated. These are pudendal (clitoris), pelvic (vagina) and hypogastric (uterus, cervix) nerves. These nerves are all genitospinal nerves – they run from the genitalia and then project into a person’s spinal cord.

In FINSEX, women were asked if they usually achieve an orgasm during sexual intercourse *via* stimulating of the clitoris, of the vagina, or both. More than half of women (54%) responded that they usually achieve an orgasm *via* stimulating both the clitoris and vagina (Fig. 9). Orgasms that result from such stimulation have been called blended orgasms (Ladas, Whipple & Perry, 2005) or fusion orgasms (Otto, 1999). A third of women (34%) reported that they usually attained an orgasm *via* stimulating the clitoris. Only 6% of women reported that they usually have an orgasm *via* stimulating the vagina. Also 6% of women told that they had never experienced an orgasm in intercourse.

The technique of how women usually stimulated their sexual organs (clitoris or vagina) had a strong association with their orgasmic capacity in intercourse (Table 2). Those women who typically experienced vaginal stimulation during intercourse had orgasms more often (64%) than did other women. Women who usually achieved orgasm *via* stimulating the clitoris achieved orgasm less frequently during intercourse (40%). In this clitoral stimulation subgroup were the biggest group of women (8% of this group) who had never had an orgasm during intercourse. This raises the question of whether a recommendation to focus mainly on clitoral stimulation in sexual intercourse is a helpful instruction to all women and their partners.

Sexual techniques that include active partner involvement are effective to female orgasmic capacity. One of these is concentrating on one’s partner for a longer time. Duration of intercourse was strongly associated with women’s ability to experience orgasm during intercourse (Table 2, *p*<0.001). Those women whose love-making usually lasted a minimum of 15 min achieved an orgasm more easily than women whose intercourse was shorter). However, if intercourse lasted for longer than 20 min, the additional effect on increasing a woman’s probability of experiencing an orgasm was marginal. An exception to this was women who experienced an increased capacity for multiple orgasms in cases of intercourse lasting for more than 1 h.

Another example of behavior that is associated with higher orgasmic frequency is the sexual position of partners in the most recent intercourse experience (results not shown in the Tables). If women were more active – including engaging in woman-on-top position, or using several positions with the partner during that intercourse – two-thirds of women achieved one or more orgasms during the intercourse. If their partner was more active, including man-on-top positions, less than half of women achieved orgasm. Sanchez, Kiefer, and Ybarra (2006) have suggested that women with an orgasm disorder tend to behave according to the traditional female scripts, in which the woman remains passive, does not let go mentally, and waits until her male partner evokes feelings of arousal and pleasure in her.

#### How partners can promote female orgasms

One way in which partners can promote female orgasms is by providing women with oral sex. Partner’s manual stimulation to female sexual organs has almost the same effect. The more frequently women receive oral or manual sex from their partners, the more often they have orgasms (Table 2, *p*<0.001). However, oral sex does not stimulate all women to achieve orgasm. Even among the women who received oral sex most of the time in sexual activity, only 60% usually achieved orgasm during intercourse, and 69% experienced orgasm in their latest intercourse.

Some women find requesting oral stimulation from their partners to be difficult. In Salisbury’s and Fisher’s study (2014), the majority of women believed that asking for, or engaging in clitoral stimulation in the presence of their male partner would not be welcome.

If a woman experienced low sexual desire, the role of oral sex in promoting orgasm was notable. Of women who very often lacked sexual desire, only around 20% experienced orgasm in their latest intercourse if their partner did not provide oral sex. 50% of low desire women, who received oral sex from their partner frequently, had an orgasm.

If women did not lack sexual desire, the role of oral sex in promoting orgasm was much less notable. Oral sex was associated with an orgasm somewhat, but even without oral sex, most of these women had orgasms in their latest intercourse. Sexual desire and related arousal were associated with female orgasms, even when sexual techniques were limited.

The role of oral sex in promoting female orgasm was notable also in couples who found it difficult to discuss of sexual issues. If discussions of sexual issues with partners were quite difficult, oral sex provided by the partner had a very significant association with women’s orgasm in their latest experience of intercourse (65% vs. 27%). It is possible that oral sex could significantly compensate for the missing sexual communication between the partners. In cases where sexual communication was open and easy, the role of oral sex in orgasms was much less remarkable (76% vs. 61%). Good sexual communication contributed to female orgasms almost as much as favorable sexual techniques.

#### The role of female sexual self-esteem and communication with the partner

Another significant factor in female orgasms was sexual self-esteem. In the ORGSEX survey, women who agreed with the statement that they were good in bed had orgasms much more frequently in comparison with women who disagreed with this statement (Table 2, *p*<0.001). Of those women who agreed completely with this statement, around 80% reported having orgasms most times in their intercourse, and as many had orgasmic experiences in their latest intercourse. If women disagreed completely that they were good in bed only 1 in 10 had had an orgasm in their latest intercourse. Orgasmic capacity is obviously one key factor by which women estimate how good they are in bed.

For some women orgasmic capacity is a learning process. Regarding their sexual self-esteem, they have learned to accept themselves and their body. They have also learned how to concentrate completely on love-making. They have often been successful in preventing stress, and to stimulate a high arousal in their intercourse. In addition, if they have had a skillful and desirable partner, they can be highly orgasmic.

In the FINSEX survey, one question asked if women agreed that they are quite skillful in sexual issues. This type of sexual self-esteem was positively associated to orgasm. Of women who agreed with this statement around 70% had an orgasm in their latest intercourse. This type of self-esteem was more strongly associated with orgasm ability than open communication regarding sexual issues with the partner (56% had orgasm).

If women did not consider themselves sexually skillful and their sexual communication with their partner was quite difficult, only about one-fifth of them experienced an orgasm in their latest intercourse. This suggests that both sexual self-esteem and communication skills with their partner are important factors that can be associated with orgasmic capacity.

In addition to sexual self-esteem, and particularly in relation to a positive assessment of sexual skills, active female sexual communication with their partner can make a big difference to orgasmic capacity. In this study, high sexual self-esteem had a very positive association with orgasms in the latest intercourse, even when sexual communication with a partner was problematic. This suggests that even in relationships that would not be considered positive, women may experience a lot of sexual pleasure if they have high sexual self-esteem. But in cases where their sexual self-esteem was low, good sexual communication with the partner significantly helped a woman’s ability to achieve an orgasm.

These FINSEX results are in line with the results of the ORGSEX survey. The ORGSEX survey included a question regarding how women had learned to enjoy love-making more intensely, and to experience orgasms. Almost half of the women reported that they had achieved this by learning to accept themselves and their body, or by concentrating completely on love-making. This may suggest that women who had felt responsible for their own pleasure had been more successful in gaining pleasure during intercourse.

Another factor that made a difference was the partner. A third of women reported that they had learned to experience orgasms by finding a desirable and skillful partner. A fifth of women had gained more orgasms by learning how to increase her partner’s pleasure. A process of mutual pleasure had presumably promoted more pleasure for women.

#### Factors that were frequently associated with a low or high probability of having orgasms

There were a number of factors that seemed to complicate female experiences of orgasms. In the ORGSEX survey a question asked ‘what prevents or inhibits respondents the most in achieving orgasm’. The most frequent responses were ‘fatigue or stress’ and ‘difficulty concentrating’. Most women selected reasons that related to their own qualifications. The next most frequent responses were ‘unskillful partner’ and ‘partner is too fast’. One-fifth of women attributed their orgasmic problems to their partners.

Based on the results of the FINSEX survey, a low frequency of female orgasm is in many ways related to the interaction with the partner. Only about a quarter of women had an orgasm in their latest intercourse if they did not consider sex at all important for the happiness in relationship; if they did not have any physical intimacy in their relationship; if they often lacked sexual desire; if their partner never had given manual or oral sex; if their intercourse was very often painful; or if their partner very often experienced an orgasm too soon.

Other factors related to low frequency of orgasm in the most recent intercourse included feeling the relationship was quite unhappy; not considering oneself sexually desirable; continuous vaginal dryness; partner’s frequent erectile problems; illness hampering sexual interaction; and intercourse that lasted at maximum only 5 min. For these women, the rate of orgasm at previous intercourse was only about one-third.

Finally, we examined the associations between various factors and female orgasmic capacity in joint regression models to see, if these factors influenced women’s ability to achieve orgasm even when we controlled the effects of other factors. Results are presented in Table 3 (for FINSEX-data) and Table 4 (for ORGSEX-data). It appears that there were a number of factors that were associated with high probability of having orgasms. These can be classified into three groups:

The first of these was related to some types of innate skills that enabled women to enjoy sexual experiences. These women had experienced their first orgasm in intercourse at a younger age than others; they did not practice active masturbation; and they had orgasms at least as easily in intercourse as in masturbation. In addition, they did not experience pain in sexual intercourse.

A second group includes factors such as good sexual skills and high sexual self-esteem. These women considered themselves good in bed. They got orgasms due to powerful arousal, and they were able to concentrate completely on love-making. They also considered it important to get orgasms in love-making and they also considered sex important for the happiness in their relationship. These women had both great mental and bodily capacity to let go and to experience orgasms.

A third group of factors relates to a woman’s skillful partner. The partner was able to promote female orgasm if he was not too fast. There was enough time to concentrate on sexual pleasure with the partner and communication with the partner was open enough in sexual issues.

Although many variables had highly statistical associations with women’s orgasmic capacity, differences in the orgasm frequency between women were not necessarily very large and in some cases diminished once age of the respondents and time of the survey was controlled for. These variables included frequency of intercourse and oral sex.

#### Multi-orgasmic women

Women show a greater propensity than men to experience multiple orgasms as a result of sexual intercourse, or other sexual stimulation. Women have not been shown to experience the same kind of post-orgasmic latent state of arousal as men do, who have just ejaculated. There is some evidence that this is probably connected to the different hormonal functions of men and women (Blackledge, 2004).

In the FINSEX survey, 12% of women reported that they had two or more orgasms in their latest intercourse, and in the ORGSEX survey 11% of women reported the same. In the ORGSEX survey, half of these multi-orgasmic women (5%) had more than two orgasms. In the FINSEX survey, these figures were relatively stable across data from 1999, 2007, and 2015. There was no increase in the proportion of multi-orgasmic women.

Almost half of the women surveyed were multi-orgasmic if they used almost continuously in their sexual activities some sexual toys and aids, or if their love making lasted for more than an hour. One-third of women were multi-orgasmic if they reported intercourse every day, or if they agreed completely that they were good in bed. One quarter of women were multi-orgasmic if they considered orgasm in intercourse very important; if they experienced sexual desire more than once a day; or if they preferred having intercourse every day. Women achieved frequent orgasms just as easily *via* masturbation as love-making.

In many respects, multi-orgasmic women displayed strong sexual interests, and were sexually very active. This goes back to the question of whether strong sexual interests resulted in these women being multi-orgasmic, or if it is a case of the very positive sexual experiences encouraging their broad spectrum of sexual appetites. There is probably no definitive answer. The only thing that can be said is that strong sexual interest and sexual enjoyment often seem to be concentrated in the same women, and this is probably comparable to men.

## Discussion

Regarding the issue of gender equality, Finland is ranked among the leading countries in the world. There has been a major improvement in gender equality since the 1970s. Social and public discourses on the subjects of gender and sexuality have underscored women’s sexual rights and the right to sexual enjoyment. Positive female sexual expectations have been increased, for example, by progress in comprehensive sexuality education; increasing sexual knowledge; and by improved sexual health services. Altogether, these were expected to enable advancing sexual pleasures to women.

Feminists in particular have assumed that improving gender equality should have a positive impact on female sexual pleasure, including orgasms. This assumption could be tested in this study by analyzing long term trends in female orgasms from the 1970s to the present time. The result was that there has been no improvement in female orgasmic capacity since the 1970s. Improving gender equality has not helped women to experience progress in this key factor of female sexual pleasure even in the 2000s. This finding is contrary to previous expectations.

Young women (under 35 years of age) have had even more difficulties in having orgasms during intercourse in the 2000s. This trend continued to the year 2015. It is a mystery why the difficulty of having female orgasms has increased in the 21st century, at a time when public information about how to better achieve orgasms proliferates. The internet and women’s- and health magazines are full of instructions regarding the pursuit and cultivation of sexual pleasure. In addition, women’s rights to sexual pleasure have been acknowledged without any doubt. That has not given any boost to improving sexual pleasure.

There have been new lifestyle- and value factors that can have limited young women’s orgasmic capacity than in preceding generations. Stressful and busy lifestyles have resulted in a lack of time; reduced strength of private life; and in increasing mental pressures that have caused difficulties to concentrate on intimate life and sexual interaction. Although there has been a parallel major increase in masturbation habits, it has not helped young women to achieve more frequent orgasms.

The findings of this study indicate that women differ greatly from one another in terms of their tendency and capacity to experience orgasms. Inequality in sexual enjoyment is much greater among women than among men. There are a number of women who are multiorgasmic, but at the same time, there are a large number of women who have never had an orgasm during intercourse. Almost half of women do not orgasm most of the time when they have intercourse. This inequality among women warrants a more comprehensive study about the predictors of female orgasms.

There have been claims that high proportions of women probably do not report their orgasms because they report, or the surveys ask about orgasms only *via* vaginal intercourse. In this framework, low orgasmic prevalence could be true, based on a limited understanding of the concept of intercourse. In this study, the concept of intercourse was adopted in the FINSEX surveys but in the ORGSEX survey, the concept adopted was love-making thus allowing respondents to decide more freely what they include in the concept. However, asking about orgasms in love-making gave exactly the same results regarding orgasmic prevalence during intercourse. Women seem to connect similar components in their mind relating to intercourse as to love-making.

The promotion of self-stimulation *via* masturbation (Komisaruk et al., 2006) is assumed, especially in sexual therapy, to contribute to an increase in female orgasms. In this study, women did not have more frequent orgasms by increasing their current practice of masturbation, or by increasing experiments with different partners in their lifetime. Orgasms did not seem to be something that could be learned *via* increasing physical experiences or *via* frequent masturbation. Women masturbate nowadays much more actively than in the 1970s, but that reform has not helped them to increase their orgasmic capacity in intercourse. On the contrary, women who had not been active in masturbation lately experienced orgasms even more regularly in their intercourse.

There has been a continuous declining trend regarding the age of first orgasm in masturbation, but not regarding the age of the first orgasm in intercourse. Nowadays, half of women have had their first orgasm in masturbation at least 5 years prior their first orgasm in intercourse. They have had more time to practice their sexual pleasure *via* masturbation before their first intercourse, but that has not helped them to achieve an orgasm any younger during intercourse. This result diverges from expectations.

There are even some findings that masturbation is associated with poorer relationship quality, greater risk of female sexual arousal disorder, impaired sexual satisfaction, impaired orgasm (especially vaginal orgasm) and with other adverse processes (Brody, 2007). In this study, female relationship quality was not associated to masturbation frequency but general sexual satisfaction was lower among women who masturbated actively. Active masturbators considered their intercourse more often very pleasant than women who masturbated less often.

Those women who had orgasms much more easily *via* masturbation had problems to experience it in intercourse. The ease of attaining an orgasm *via* masturbation was not a good measure of orgasmic capacity during intercourse. Half of the women surveyed usually had an orgasm in intercourse *via* stimulating both clitoris and vagina, and only one-third usually *via* stimulating clitoris. Based on these results, the role of the clitoris is not as dominant in sexual stimulation towards orgasm in intercourse as has been expected.

Meston, Hull, Levin, and Sipski (2004) have argued that there are no consistent, empirical findings that psycho-social factors alone differentiate orgasmic from anorgasmic women. This study can add more information about how these factors are actually associated with more- or less frequent orgasms. These factors include women’s sexual desire, sexual values, sexual self-esteem, skills in communication, and their partner’s qualities.

The keys to achieving more frequent female orgasms were identified in this study as being in the mind and in the relationship. These factors and capacities included how important orgasms were considered personally; how high was sexual desire; how high was sexual self-esteem; and how open was sexual communication with the partner. Sexual self-esteem included how sexually skillful and how good in bed women considered themselves. Other positive factors of orgasmic capacity were the ability to concentrate on the moment; mutual sexual initiations; and a partner’s good sexual techniques. All of these are factors that should be paid attention to in sexual therapy.

Based on regression analysis, women who had a high orgasmic capacity had an innate talent to react with arousal to sexual stimulation in intercourse. They were also sexually skillful and they had good sexual self-esteem. Thanks to their good sexual experiences, they valued orgasms in their intercourse and in their relationship. Very often they also had a skillful sexual partner, who provided them good stimulation and was able to discuss sexual issues openly. They were able to maintain a positive circle that even increased their orgasmic capacity.

In short, a relationship that felt good and worked well emotionally, and where sex was approached openly and appreciatively, was associated with orgasmic capacity. These same factors were even more pronounced among multi-orgasmic women. In addition to this, they realized more frequent and long-lasting love-makings and sexual role plays. Thanks to their highly pleasurable intercourse, they had a high and versatile sexual motivation.

Although masturbation has not stimulated women to achieve more frequent orgasms, they surely need a variant sexual stimulation to become aroused during sexual intercourse, and also to have an orgasm. An orgasm is a complex response to socially contextualized physical and mental stimuli and, for the individual, there will be a variety of sources of effective stimulation, both physically and mentally (Lauman, Gagnon, Michael & Michaels, 1994). Based on ideas of Levin (2014), the factors that are involved in influencing the pleasure of orgasms are the novelty of sexual stimulation; genital stimulation with concomitant stimulation in and around the anus; the use of sexual fantasy; the duration between orgasms; and the duration and expertise in the sexual stimulation leading up to the climax are all reasonably well-known enhancing behaviors.

In this same spirit, Paterson, Jin, Amsel, and Binik (2014) have found that a greater build-up of sexual arousal desire prior to orgasm significantly predicted orgasmic pleasure for both genders. They recommend enhancing the experience of orgasm by delaying it until having reached high levels of sexual arousal and desire. According to Adam, Génet, Day, and Sutter (2015) orgasmic women reported significantly more mindfulness (they were capable of concentrating) during dyadic sexual activities. By the same token, Laan and Rellini (2011) argue that women’s orgasm consistency in all forms of partnered sexual activity is associated with sexual autonomy. In addition, letting go of control is mandatory for an orgasm to take place (Georgiadis et al., 2006).

Teaching effective techniques of stimulation may well improve the orgasmic response of anorgasmic women (McCabe, 2009). Ignorance of the best techniques; reluctance to use them; and/or an inability to communicate preferences for sexual stimulation to the partner contribute to low orgasmic frequency during sexual interaction (McCabe, 2009).

Based on the results of this study, one key issue regarding female orgasmic capacity is a personal sexual motivation. Women who have a high sexual motivation; desire to have sex; communicate sexual issues openly with their partner; make sexual initiations; and are active in sexual intercourse are more likely to experience orgasms during intercourse. Sexual activity means, for example, using woman-on-top positions in intercourse, and providing pleasure to the partner. Activity in sexual communication helps women to get the kind of stimulation that they need for arousal towards an orgasm from their partner.

Many studies have reported the importance of good sexual communication. Empirical studies have consistently demonstrated that anorgasmic women reported experiencing significantly greater discomfort with communication about sexual activities (Kelly, Strassberg & Turner, 2004). A lack of communication between partners about their sexual relationship appears to be a factor related to anorgasmia in women (McCabe, 2009).

Past failure to achieve orgasm can elicit self-defeating and distracting thoughts about whether a woman will be able to achieve orgasm this time. A woman may mentally monitor her own- and her partner’s response, unable to allow herself to relax and enjoy the sexual stimulation for its own sake. She becomes a spectator who demands her body’s response (McCabe, 2009). When someone is not fully engaged in love-making, it is no surprise that the enjoyment it brings is not what it could be.

Public discourses and social expectations in today’s society have mental implications for women and for their capacity for pleasure. The persistent risk discourse that relates to sexual issues may have created a situation in which women increasingly view sexual interaction through a more rational lens, rather than casting their body and soul into enjoying sexual experiences with a partner and realizing their own desires. Excessive rationalism is the biggest enemy of orgasms. Simply put, thinking does alight desire, but orgasms come when thinking ceases. The inability to implement this formula may be one key issue that particularly young women are increasingly experiencing regarding orgasms.

One outcome of female infrequent orgasms can be their lower sexual desire in comparison to males. In the spirit of the social exchange theory (Sprecher, 1998), one could think that the greater rewards that men achieve on average from sexual interaction could explain their stronger sexual desires. This might make sense, in that men experience orgasms so much more often and more easily in intercourse compared to women. In other words, men might be more motivated to seek intercourse as something that offers them particular rewards. This viewpoint was supported also by the finding that women who enjoyed sexual intercourse, and got more pleasure out of their sex lives than other women, were also less likely to experience lack of sexual desire. If women were to enjoy intercourse more and have orgasms more regularly, the desire gap between the genders should decline.

This study has its limitations. The wording of the items could be more exact. There could be other items that would measure more comprehensively the predictors of female orgasms. One example is differentiating between vaginal and clitoral orgasm. They have different psychological pathways and processes (Brody & Costa, 2009). In addition, this study does not enable causal explanations between the predictors and the dependent variable, the frequency of female orgasm. There is a need for a longitudinal study. Some of the trends and associations could be better understood by conducting qualitative studies.

One more challenge for future studies is to understand why a great number of women value their partner’s orgasm much more than their own. According to their responses, women’s sexual behavior includes quite often altruistic components. Women assumingly would value their own orgasms more if they would get them more easily and more frequently. Sexual pleasure can increase female sexual motivation. By actively promoting female orgasms, we could create a positive circle that would favorably increase female sexual pleasure.
